# Trajectories of physical performance in nursing home residents with dementia

**DOI:** 10.1007/s40520-020-01499-y

**Published:** 2020-02-14

**Authors:** Karen Sverdrup, Sverre Bergh, Geir Selbæk, Jūratė Šaltytė Benth, Irene M. Røen, Bettina Husebo, Gro G. Tangen

**Affiliations:** 1 Norwegian National Advisory Unit On Ageing and Health, Vestfold Hospital Trust, Tønsberg, Norway; 2 Department of Geriatric Medicine, Oslo University Hospital, Oslo, Norway; 3 Department of Interdisciplinary Health Sciences, Institute of Health and Society, Faculty of Medicine, University of Oslo, Oslo, Norway; 4 Research Centre for Age-Related Functional Decline and Disease, Innlandet Hospital Trust, Ottestad, Norway; 5 Institute of Clinical Medicine, Faculty of Medicine, University of Oslo, Oslo, Norway; 6 Institute of Clinical Medicine, Campus Ahus, University of Oslo, Oslo, Norway; 7 Health Services Research Unit, Akershus University Hospital, Lørenskog, Norway; 8 Centre for Development of Institutional and Home Care Services, Hamar, Hedmark Norway; 9 Department of Global Public Health and Primary Care, Centre for Elderly and Nursing Home Medicine, University of Bergen, Bergen, Norway; 10 Municipality of Bergen, Bergen, Norway

**Keywords:** Physical performance, Trajectories, Dementia, Nursing home

## Abstract

**Background:**

In nursing homes (NH) the prevalence of dementia ranges from 50 to 84% and most residents have extensive physical-performance impairments. However, from time of admission, development of physical performance in NH residents with dementia remains unexplored.

**Aims:**

To explore the overall trend in physical performance, associated characteristics, and groups following distinct trajectories from time of admission, in NH residents with dementia.

**Methods:**

We followed newly admitted NH residents diagnosed with dementia (*N* = 583) from 47 NHs across Norway for 3 years. Individual assessments were conducted biannually, and main outcome measure was the Short Physical Performance Battery (SPPB). Facility-level characteristics included unit size, staff-to-resident ratio, and quality of the physical environment (Special Care Unit Environmental Quality Scale, SCUEQS).

**Results:**

From time of admission, NH residents with dementia showed a significant overall decline in physical performance. Further, we identified three distinct trajectory groups with significantly different baseline physical-performance status (“good,” “moderate,” and “poor”), differences between groups maintained and all declined across time. Younger age, good general medical health, less-severe dementia, and less musculoskeletal pain were associated with both an average higher overall trend and better baseline group-belonging. Additionally, less apathy and more psychosis were associated with a higher overall trend, and agitation was associated with poorer baseline group-belonging.

**Conclusions:**

To prevent excessive decline in physical performance in this population, NH clinicians should focus efforts specifically on assessment of physical performance at admission and on identification and management of musculoskeletal pain and neuropsychiatric symptoms.

**Electronic supplementary material:**

The online version of this article (10.1007/s40520-020-01499-y) contains supplementary material, which is available to authorized users.

## Introduction

Increased life expectancy is one of humanity’s greatest triumphs. However, as the proportion of older adults continues to grow, the number of dependent older adults worldwide also increases [1]. This will directly influence the demand for nursing home (NH) care [1]. Further, in 2015, over 46 million people lived with dementia globally, a number expected to increase to 131.5 million by 2050 [2]. Dementia and physical performance impairments are major predictors for NH admission [3]. In NHs the prevalence of dementia ranges from 50 to 84% [1, 4] and most residents have extensive physical-performance impairments [5, 6].

Impairments in cognitive and physical performance often co-occur in older adults [7, 8]. People with dementia exhibit a greater decline in physical performance compared to cognitively healthy adults [9], and as dementia severity increases, physical performance progressively deteriorates [10]. However, this relationship is not as clear in NH residents as in community-dwelling older adults [5].

A recent narrative review showed an overall declining trend in physical performance over time in NH residents [11]. However, the included studies were mainly intervention trials, generally applying narrow inclusion criteria and often excluding residents with dementia. Further, none of these studies examined changes in physical performance from time of admission to the NH. Within the physical-performance limitations observed in NH residents, they are still described as heterogeneous [5, 12]. This heterogeneity remains unexplored in a longitudinal design.

In addition to impairments in cognitive and physical performance, most people with dementia also experience neuropsychiatric symptoms (NPS) and pain [13–15]. Further, the importance of facility-specific characteristics in NHs is increasingly recognized as a key component in caring for residents with dementia [16], and the quality of the physical environment itself may influence function [17, 18].

Physical performance is a prerequisite for mobility and successful completion of activities of daily life (ADL), and impairments significantly influence quality of life for individuals affected in addition to imposing increased risks for disability, dependency, and mortality [19–21]. To the authors’ best knowledge, no previous studies have explored the development of physical performance over time and associated characteristics, in nursing home (NH) residents with dementia and attaining this knowledge is deemed essential.

Therefore, the aims of this study were to explore the overall trend in physical performance and individual and facility-specific characteristics associated with it, to identify groups with distinct trajectories, and to assess baseline characteristics associated with group-belonging, from time of admission, in NH residents with dementia.

## Methods

### Design, procedure and participants

This multicenter prospective study is based on data from the Resource Use and Disease Course in dementia-Nursing Home (REDIC-NH) study [4]. Data were collected from 47 NHs across 35 municipalities in Norway by NH health workers in collaboration with research nurses. Residents were consecutively included at admission to the NHs; baseline data were collected within one month of admission between March 2012 and November 2014. Follow-up data were collected every 6 months for 36 months, with the last data collected in May 2017.

Participation was based on consent given by the resident or the resident’s next of kin when the resident was unable to consent. Ability to give consent was decided by the NH staff, including the physician, in collaboration with the resident’s next of kin. The study was approved by the Regional Ethics committee for Medical Research in South-Eastern Norway (2011/1738a).

All residents 65 years and older were eligible for inclusion. Additionally, residents younger than 65 who had an established dementia diagnosis were included. Residents with an expected stay of less than four weeks or life expectancy less than six weeks, were excluded. In total, 696 residents were included in REDIC-NH. At baseline, dementia was diagnosed according to the International Classification of Diseases, version 10, research criteria (ICD-10) [22] independently by two old age psychiatrists (SB and GS). When no consensus was reached, a third old age psychiatrist was consulted. In all, 583 residents were diagnosed with dementia and included in the present study.

### Measures

#### Individual characteristics

Individual characteristics include age, sex, years of education, number of medications, general medical health, physical performance, dementia severity, pain, and neuropsychiatric symptoms (NPS). Number of medications, based on counts of the Anatomical Therapeutic Chemical (ATC) Classification System for coded medications, was collected from the NH records. General medical health was assessed with the General Medical Health Rating (GMHR) scale, a one-item global rating scale with four categories (poor, fair, good, excellent) [23]. We dichotomized GMHR into excellent/good versus fair/poor.

Residents’ physical performance was assessed with the Short Physical Performance Battery (SPPB) [24]. The SPPB is a performance-based test that comprises three components: a hierarchical balance test, a 4-m walking test, and a 5-times chair-stand test. Each component is scored from 0 to 4 to generate a total score 0–12. A higher score indicates better physical performance [25].

The Clinical Dementia Rating (CDR) was used to assess dementia severity. The CDR is a global rating scale covering six domains of cognitive and functional performance [26]. The CDR sum of boxes (CDR-sob) is calculated by adding the domain scores (0–18), where a higher score indicates more-severe dementia [27].

Pain was assessed with the Mobilization-Observation-Behaviour-Intensity-Dementia Pain Scale (MOBID-2) [28]. The MOBID-2 is a two-part observational instrument with five items; scoring is from 0 (no pain) to 10 (as bad as it could possibly be) in each item. Part 1 (0–50) assesses pain related to the musculoskeletal system; Part 2 (0–50) assesses pain related to internal organs, head, and skin. Higher score indicates greater pain.

NPS were assessed using the 12-item Neuropsychiatric Inventory-Nursing Home Version (NPI-NH), which assesses frequency and severity of 12 NPS [29]. NPI sub-syndrome scores were calculated based on a previous principal component analysis [30] and include NPI agitation (agitation/aggression, disinhibition, irritability, 0–36), psychosis (delusions and hallucinations, 0–24), affective (depression and anxiety, 0–24), and apathy (apathy, 0–12).

#### Facility-specific characteristics

Facility-specific characteristics measuring unit size (number of residents) and staff-to-resident ratio (number of NH staff per resident during daytime hours) were collected through questionnaires and interviews with NH managers. The quality of the physical environment was assessed with the Special Care Unit Environmental Quality Scale (SCUEQS) [31]. The SCUEQS comprises 18 items measuring maintenance, cleanliness, safety, lighting, physical appearance/homelikeness, orientation/cueing, and noise. Composite scores range from 0 to 41; higher scores indicate better physical environment. The SCUEQS data were collected through structured observations and interviews between October 2013 and November 2014. The process has been published in detail previously [32].

### Statistical analysis

Missing values on CDR, MOBID-2 Part 1 and 2, and NPI-NH were imputed for cases with fewer than 50% missing items. The empirical distribution for each item was generated, and random values drawn from it were used to replace missing values. If one of the three item scores were missing in SPPB, the total score was calculated as the sum of the two non-missing scores plus their average [33]. Characteristics of residents and NHs were described as means and standard deviations (SD) or as frequencies and percentages.

To explore the overall trend in physical performance (SPPB) throughout the study period and characteristics associated with it, a linear mixed model (LMM) was estimated. First, the model with fixed effects for non-linear time was estimated. Then, individual and facility-specific characteristics, one at a time, were included as additional fixed effects in the model, together with the interaction between time and characteristic. Finally, a multiple model with all characteristics and corresponding interactions was estimated and reduced for excessive interactions by applying Akaike’s Information Criterion (AIC). All models contained random effects for residents nested within NH units.

A growth mixture model (GMM) was estimated to identify potential groups of residents following distinct trajectories in physical performance (SPPB) throughout the study period. Cases with missing physical performance (SPPB) information at baseline were excluded from the analyses. The number of groups was determined by applying the log form of the Bayes factor, interpreted as the degree of evidence favoring the alternative model [34]. Also required were reasonable group sizes, that 95% confidence intervals (CI) of trajectories were non-overlapping, and that within-group probabilities were at least 0.80. Next, nominal regression analysis for hierarchical data was performed to assess whether individual and facility-specific characteristics measured at baseline were associated with group-belonging. The model contained random effects for NH units to account for within-unit correlations.

Only cases with no missing values on covariates were included in the regression analyses. Both LMM and GMM model handle unbalanced data sets by incorporating all available information, also from dropouts, in the model estimation. Statistical analyses were performed using IBM SPSS V25, SAS V9.4 and STATA V14. Results with *p* values below 0.05 were considered statistically significant.

## Results

At admission to the NH, the average age of residents with dementia was 84.1 years (SD 7.5) and 35.5% were men (Table 1). Of the 583 residents at baseline, 170 were assessed after 36 months; attrition was due mainly to death (*N* = 353) (Table S1).

**Table 1:** Characteristics at baseline, *N* = 583

Characteristic	Statistic
Age, mean (SD)	84.1 (7.5)
Sex, male, *n* (%)	207 (35.5)
Education (years), *n*/mean (SD)	428/8.3 (2.9)
SPPB, *n*/mean (SD)	531/4.3 (3.6)
GMHR, *n*/*n* Good (%)	557/277 (49.7)
Medication, mean (SD)	5.7 (3.1)
CDR-sob, *n*/mean (SD)	578/11.3 (3.6)
MOBID-2 Part 1, *n*/mean (SD)	568/4.8 (6.5)
MOBID-2 Part 2, *n*/mean (SD)	567/3.5 (4.8)
NPI psychosis, *n*/mean (SD)	581/1.9 (4.1)
NPI agitation, *n*/mean (SD)	581/4.5 (7.3)
NPI affective, *n*/mean (SD)	581/3.9 (5.9)
NPI apathy, *n*/mean (SD)	582/1.3 (2.7)
SCUEQS, *n*/mean (SD)	564/25.3 (4.7)
Unit size, mean (SD)	10.8 (4.8)
Staff-to-resident ratio, mean (SD)	0.32 (0.1)

*SD* standard deviation, *SPPB* the short physical performance battery (0–12), *GMHR* general medical health rating (dichotomized excellent/good versus fair/poor), *CDR-sob* clinical dementia rating sum of boxes (0–18), *MOBID-2* mobilization-observation-behaviour-intensity-dementia pain scale part 1 (0–50) and part 2 (0–50), *NPI* neuropsychiatric inventory psychosis (delusions, hallucinations, 0–24), agitation (agitation/ aggression, disinhibition, irritability, 0–36), affective (depression, anxiety, 0–24) and apathy (apathy, 0–12), *SCUEQS* special care unit environmental quality scale (0–41)

### Overall trend in physical performance and associated characteristics

**Fig. 1:**
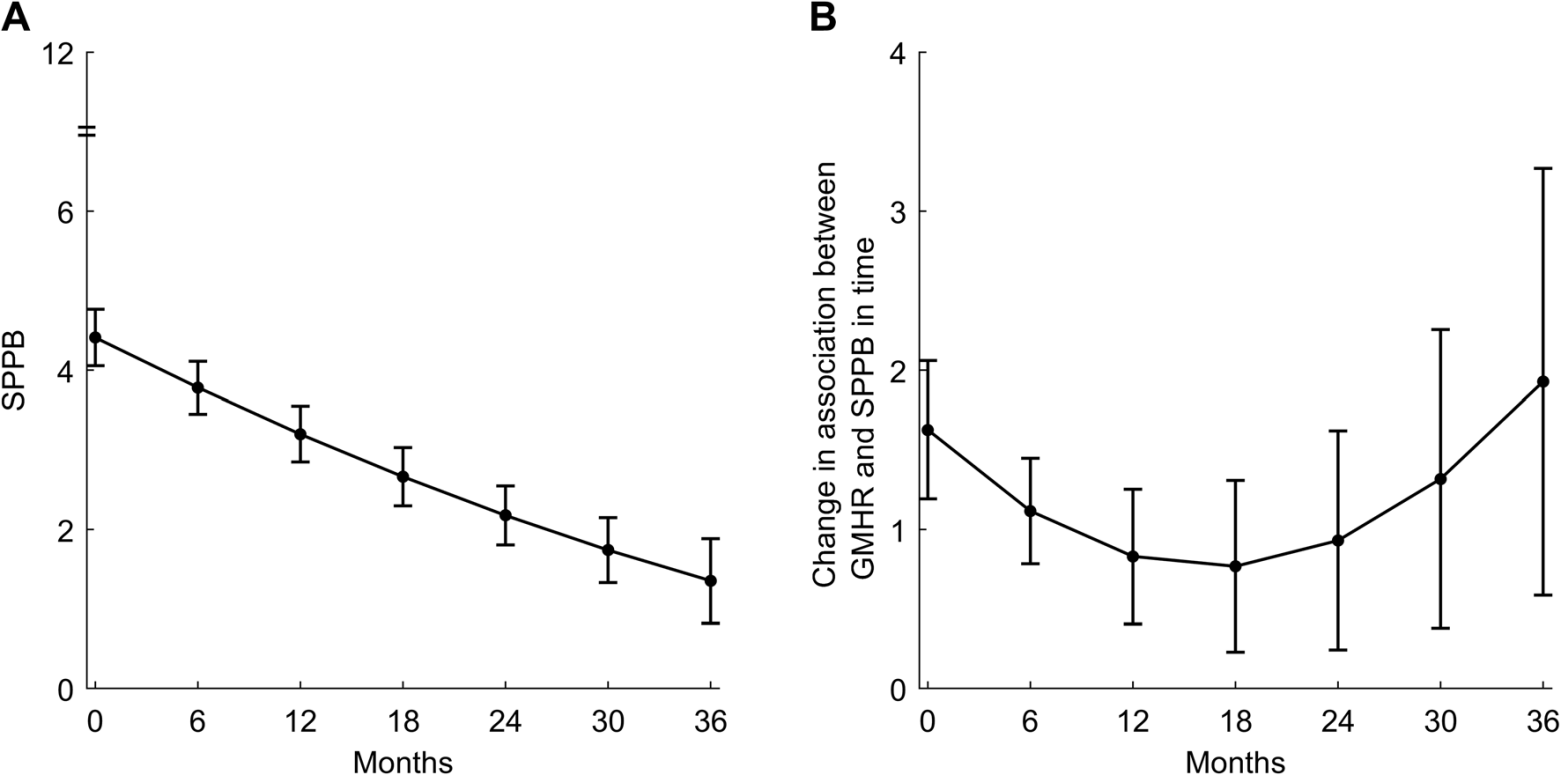
Unadjusted overall trend in physical performance (SPPB) (**a**) and change in association in time between SPPB and GMHR (**b**) over 36 months of follow-up. *SPPB* short physical performance battery (0–12), *GMHR* general medical health rating (dichotomized excellent/good versus fair/poor)

In the unadjusted model, the overall trend in physical performance (SPPB) declined significantly throughout the study period, from 4.4 (baseline) to 3.2 (12 months), 2.2 (24 months), and ending at 1.4 after 36 months (Table S2, Fig. 1a). In the multiple model, younger age (*p* < 0.001), less-severe dementia (CDR-sob, *p* < 0.001), less musculoskeletal pain (MOBID-2 Part 1, *p* < 0.001), more psychosis (NPI psychosis, *p* = 0.02), and less apathy (NPI apathy, *p* = 0.02) were associated with an average higher overall physical performance (SPPB) throughout the study period (Table S2).

Overall, there were differences in trend in physical performance (SPPB) between residents with poor and residents with good general medical health (GMHR) throughout the study period (significant interaction terms). Good (GMHR) was associated with higher physical performance (SPPB) throughout the study period (*p* < 0.001), although with association varying with time. The association between general medical health and physical performance (SPPB) was found to decrease slightly in the beginning of the follow-up and increase after 24 months (Table S2, Fig. 1b).

### Trajectories of physical performance

**Fig. 2:**
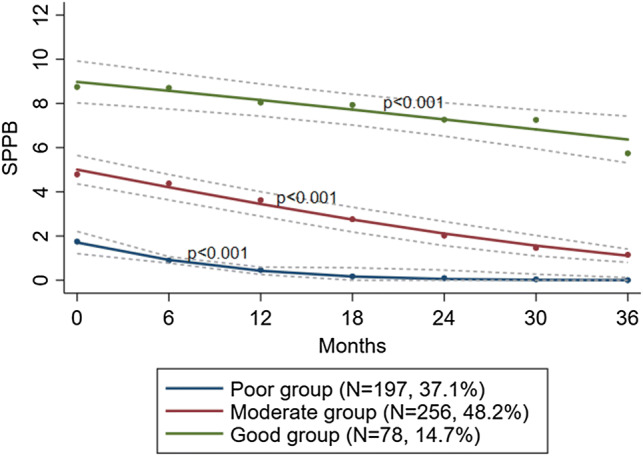
Trajectories of physical performance (SPPB) with 95% confidence intervals over 36 months of follow-up by group-belonging. *SPPB* short physical performance battery (0–12)

We identified three distinct groups labeled “Good” (*n* = 78, 14.7%), “Moderate” (*n* = 256, 48.2%), or “Poor” (*n* = 197, 37.1%), according to baseline status (Fig. 2). Average within-group probabilities were all above 0.8. All three groups differed significantly at baseline, starting at about 9.3, 5.0, and 1.7 (SPPB), their trajectories were non-overlapping, and exhibited a significant decline over 36 months with maintained differences between groups. Table 2 shows residents’ baseline characteristics within groups.

**Table 2:** Characteristics within groups of physical performance (SPPB), *N* = 531

Characteristics	Poor (*N* = 197)	Moderate (*N* = 256)	Good (*N* = 78)
Age, mean (SD)	84.8 (7.6)	84.6 (7.1)	80.9 (7.5)
Sex, male, *n* (%)	74 (37.6)	91 (35.5)	22 (28.2)
Education (years), *n*/mean (SD)	139/8.1 (2.8)	197/8.5 (3.1)	56/8.2 (2.9)
GMHR, *n*/*n* Good (%)	187/65 (34.8)	244/132 (54.1)	76/59 (77.6)
Medication, mean (SD)	5.9 (3.1)	5.8 (3.1)	4.9 (2.9)
CDR-sob, *n*/mean (SD)	195/12.2 (3.4)	255/10.7 (3.6)	78/10.2 (2.9)
MOBID-2 Part 1, *n*/mean (SD)	193/7.0 (7.8)	252/3.9 (5.4)	78/2.0 (3.4)
MOBID-2 Part 2, *n*/mean (SD)	193/4.4 (5.2)	251/3.0 (4.4)	78/2.6 (4.2)
NPI psychosis, *n*/mean (SD)	195/2.2 (4.8)	256/1.4 (3.5)	78/2.2 (3.4)
NPI agitation, *n*/mean (SD)	195/5.8 (8.5)	256/3.2 (5.6)	78/4.5 (7.1)
NPI affective, *n*/mean (SD)	195/4.2 (6.1)	256/3.1 (5.2)	78/4.5 (6.1)
NPI apathy, *n*/mean (SD)	196/2.0 (3.4)	256/1.0 (2.2)	78/0.9 (2.4)
SCUEQS, *n*/mean (SD)	188/24.7 (4.4)	248/25.5 (4.7)	78/26.1 (4.5)
Unit size, mean (SD)	11.5 (5.1)	10.3 (4.2)	10.5 (5.1)
Staff-to-resident ratio, mean (SD)	0.32 (0.08)	0.32 (0.09)	0.31 (0.06)

Only residents with no missing SPPB at baseline included

*SPPB* short physical performance battery, *GMHR* general medical health rating (dichotomized excellent/good versus fair/poor), *CDR-sob* clinical dementia rating sum of boxes (0–18), *MOBID-2* mobilization-observation-behaviour-intensity-dementia pain scale part 1 (0–50) and part 2 (0–50), *NPI* neuropsychiatric inventory psychosis (delusions, hallucinations, 0–24), agitation (agitation/ aggression, disinhibition, irritability, 0–36), affective (depression, anxiety, 0–24) and apathy (apathy, 0–12), *SCUEQS* special care unit environmental quality scale (0–41)

In the multiple model, younger age at baseline was associated with higher odds of being in the Good compared to the Poor group (*p* = 0.001), and less agitation (NPI agitation, *p* = 0.01) was associated with higher odds of being in the Moderate compared to the Poor group. Good general medical health (GMHR Good), less-severe dementia (CDR), and less musculoskeletal pain (MOBID-2 Part 1) at baseline were associated with higher odds of being in the Moderate (*p* = 0.006, *p* = 0.007, and *p* = 0.001) and Good (*p* < 0.001, *p* < 0.001, and *p* < 0.001) groups compared to the Poor group (Table S3).

## Discussion

To the authors’ best knowledge, this study is the first to explore the overall trend in physical performance, associated characteristics, and groups following distinct trajectories from time of admission in NH residents with dementia. Our study showed a significant overall decline in physical performance over three years, and we identified three distinct groups of decline labeled “Good”, “Moderate”, or “Poor”, according to baseline physical performance status. Younger age, good general medical health, less-severe dementia, and less musculoskeletal pain were associated with an average higher overall trend and better baseline group-belonging. Additionally, less apathy and more psychosis were associated with a higher overall trend, and agitation was associated with poorer baseline group-belonging.

This study shows how physical performance substantially decrease from time of admission, in NH residents with dementia, extending the current literature from a recent narrative review [11]. Further we also quantified the decline of one point per year for three years from 4.4 to 1.4, extending the previous knowledge further than one-year follow-up [35]. A change of one point on the SPPB is recognized as a clinically meaningful change [36, 37] and a drop from 4.4 to 1.4 is considered a substantial loss of function.

In our study, pain related to the musculoskeletal system was negatively associated with physical performance, adding to previous knowledge that pain is associated with ADL function in community-dwelling older adults with dementia [38]. Assessment and treatment of pain in people with dementia is complex [28]. However, our study discriminates between musculoskeletal and internal-organ pain, whereas musculoskeletal pain and not internal-organ pain was associated with physical performance, supporting our findings. Additionally, apathy was negatively associated with physical performance, which extends the current knowledge that apathy is associated with dependency in ADL in NH residents [39]. Lack of motivation, taking initiative, and goal-directed behavior are manifestations of apathy; thus, the observed negative association between apathy and physical performance is reasonable. Further, we found a positive association between psychosis and physical performance. Previously, these symptoms have been associated with poorer clinical outcomes [40]. Our result is a novel finding requiring further investigation to elucidate. We also observed that age, general medical health, and dementia severity were negatively associated with physical performance, associations well known across populations [5, 7–9, 41].

In our study, the association between the quality of the physical environment and overall physical performance was not statistically significant (*p* = 0.051). This negative finding contrasts with previous studies of ADL function in NH residents [17, 18]. Physical environment is an easily modifiable characteristic of importance when designing the structure of NH facilities, and the near-significance of our results calls for more studies examining this association.

Another main finding was the identification of three groups with distinct, but parallel trajectories, all showing significant decline over time. A recent study of trajectories of ADL in NH residents identified different groups, which also included periods of stability or recovery [42]. However, this study was not specific to residents with dementia. In our study, and across populations, cognitive impairment is associated with decline in physical performance [5, 7–9, 41], and cognitive disorders have been shown to negatively affect trajectories of physical performance in late life [43]. Thus, our sample of residents with dementia might be particularly vulnerable to physical-performance decline, and a reason our study showed decline in all three groups. Further, mortality in REDIC-NH is high and lower function in ADL is associated with higher mortality [44]. Additionally, disability and mortality increase with decline in physical performance [24, 25] and future studies should explore this interrelationship further in the NH population.

Although all three groups experienced decline in physical performance over time, they significantly differed according to baseline physical-performance status and these differences maintained across time. The existence of significantly different levels of physical performance at admission helps to disentangle some of the heterogeneity of physical performance observed in cross-sectional studies [5, 12]. Further, it supports previous research showing that assessment of physical performance at admission is key to facilitating the provision of tailored services and care [5, 45]. The same characteristics associated with overall trend, i.e. age, general medical health, dementia severity, and musculoskeletal pain at admission, were also associated with trajectory group-belonging. Further, less agitation at admission was associated with higher odds of being included in the Moderate group compared to the Poor group. Agitation is more common as dementia severity increases [46, 47] and with decreasing levels of physical activity [46, 48]. In the Poor group, level of physical performance was low, and group-belonging was associated with both dementia severity and more agitation. Agitation expressed as aggression, inhibition, and restlessness is burdensome for the person and poses significant challenges for staff [47]. Individual comprehensive assessment and symptom management are deemed essential for this particularly vulnerable group.

Our study has several strengths including the broad inclusion criteria, wide assessment battery, standardized training of all assessors, and inclusion of residents from different NHs in a large geographical region including urban and rural areas. Further, recruitment at NH admission, follow-up assessments at equal time intervals and using a performance-based measure of physical performance contributes to the study’s strength and originality. However, some limitations should also be considered. Due to a large amount of missing information on education and, therefore, loss of a substantial number of residents, education was not included as a covariate in the regression models. When included, it did not affect the overall trend, number of distinct groups, or shape of trajectories identified. Facility-specific characteristics were assessed only once, and unit size and staff-to-resident ratios might change over time. However, we consider the physical environment as stable. Further, several characteristics were not measured, such as type of medications and specific diseases and comorbidities; these could have influenced physical performance. Further, dementia diagnosis was set without clinical examination of residents. Therefore, misclassification of dementia is possible. NHs were not selected for participation randomly but by convenience sampling. Additionally, description of the REDIC-NH study revealed that, among the included residents, there were more women than in the eligible but not included residents [4] and lack of information about generalizability is a limitation.

Taken together, musculoskeletal pain and NPS emerged as potentially modifiable characteristics associated with both overall physical performance and trajectory group-belonging. Pain and NPS are highly correlated in people with dementia, and interventions to reduce one may also reduce the other [15, 38]. Non-pharmacological interventions are recommended for managing both pain and NPS [15, 49], and because of the observed association with physical performance in this study, such interventions combined with comprehensive assessment and management may prevent the substantial observed decline in physical performance. Increased physical activity may also provide positive effects on pain and NPS [1] and individual physical-performance assessment at admission; facilitating the ability to provide tailored physical activities emerges as important.

## Conclusions

From time of admission, NH residents with dementia showed a substantial decline in physical performance and we identified three distinct groups. The groups significantly differed according to baseline physical-performance status, differences maintained, and all trajectories declined over time. Modifiable characteristics associated with both overall trend and group-belonging included musculoskeletal pain and neuropsychiatric symptoms. To prevent excessive decline in physical performance in this population, NH clinicians should focus efforts specifically on assessment of physical performance at admission and on identification and management of musculoskeletal pain and neuropsychiatric symptoms.

## Electronic supplementary material

Below is the link to the electronic supplementary material.

Supplementary file1 (DOCX 35 kb)
