# Supplementary material for: Trajectories of physical performance in nursing home residents with dementia
Source: Aging Clin Exp Res. 2020 Feb 14;32(12):2603–10. doi: 10.1007/s40520-020-01499-y (PMC7680334; doi:10.1007/s40520-020-01499-y)
Supplement: Supplementary file 1 — Supplementary file1 (DOCX 35 kb) [file 40520_2020_1499_MOESM1_ESM.docx]

**SUPPLEMENTARY MATERIAL**

| **Supplementary Table S1** Number of residents at each assessment | | | | | | | | |
| --- | --- | --- | --- | --- | --- | --- | --- | --- |
|  | **T_0_** | **T_6_** | **T_12_** | **T_18_** | **T_24_** | **T_30_** | **T_36_** | **TOTAL** |
| **Number assessed** | 583 | 437 | 374 | 307 | 260 | 209 | 170 |  |
| **Number left:** |  |  |  |  |  |  |  |  |
| Due to death |  | 84 | 65 | 60 | 54 | 42 | 48 | 353 |
| Other reasons |  | 30 | 16 | 2 | 2 | 5 | 3 | 59 |
| Missing/ Unknown |  | 32 | 14 | 18 | 9 | 13 | 4 |  |
| **Left from T_6_** |  |  | 114 | 114 | 114 | 114 | 114 | 114 |
| **Left from T_12_** |  |  |  | 81 | 81 | 81 | 81 | 81 |
| **Left from T_18_** |  |  |  |  | 63 | 63 | 63 | 63 |
| **Left from T_24_** |  |  |  |  |  | 56 | 56 | 56 |
| **Left from T_30_** |  |  |  |  |  |  | 47 | 47 |
| **Left from T_36_** |  |  |  |  |  |  |  | 51 |

| **Supplementary Table S2** Linear mixed model with physical performance (SPPB) as dependent variable, N=1666^a^ | | | | |
| --- | --- | --- | --- | --- |
| **Variable** | **Bivariate model** | | **Multiple model** | |
|  | **Regr.coeff.**  **(SE)** | **p-value** | **Regr.coeff. (SE)** | **p-value** |
| **Intercept** | 4.4 (0.20) | <0.001 | 10.4 (1.72) | <0.001 |
| **Time** | -0.11 (0.02) | <0.001 | 0.02 (0.02) | 0.40 |
| **Time x Time** | 0.0007 (0.0005) | 0.19 | -0.001 (0.0006) | 0.15 |
|  |  |  |  |  |
| **Age** | -0.08 (0.02) | *<0.001* | -0.06 (0.02) | *<0.001* |
| Age x Time | 0.0005 (0.002) | 0.83 |  |  |
| Age x Time x Time | 0.000001 (0.00007) | 0.90 |  |  |
| **Sex** (male) | -0.35 (0.31) | 0.25 | -0.38 (0.23) | 0.10 |
| Sex x Time | 0.02 (0.04) | 0.64 |  |  |
| Sex x Time x Time | -0.0006 (0.001) | 0.58 |  |  |
| **GMHR** (good) | 1.92 (0.22) | *<0.001* | 1.63 (0.22) | *<0.001* |
| GMHR x Time | -0.10 (0.03) | *0.005* | -0.10 (0.03) | *0.002* |
| GMHR x Time x Time | 0.003 (0.001) | *0.006* | 0.003 (0.001) | *0.003* |
| **Medication** | -0.09 (0.04) | *0.02* | -0.02 (0.03) | 0.51 |
| No of medication x Time | 0.01 (0.005) | *0.02* |  |  |
| No of medication x Time x Time | -0.0003 (0.0002) | *0.04* |  |  |
| **CDR-sob** | -0.11 (0.03) | *0.001* | -0.16 (0.02) | *<0.001* |
| CDR-sob x Time | -0.01 (0.005) | *0.03* |  |  |
| CDR-sob x Time x Time | 0.0001 (0.0001) | 0.43 |  |  |
| **MOBID-2 Part 1** | -0.15 (0.02) | *<0.001* | -0.08 (0.01) | *<0.001* |
| MOBID-2 part 1 x Time | 0.006 (0.002) | *0.01* |  |  |
| MOBID-2 part 1 x Time x Time | -0.0001 (0.00007) | 0.08 |  |  |
| **MOBID-2 Part 2** | -0.08 (0.03) | *0.003* | -0.002 (0.02) | 0.90 |
| MOBID-2 part 2 x Time | 0.001 (0.004) | 0.80 |  |  |
| MOBID-2 part 2 x Time x Time | -0.00002 (0.0001) | 0.89 |  |  |
| **NPI psychosis** | 0.01 (0.03) | 0.66 | 0.05 (0.02) | *0.02* |
| Psychosis x Time | -0.003 (0.004) | 0.51 |  |  |
| Psychosis x Time x Time | 0.00004 (0.0001) | 0.75 |  |  |
| **NPI agitation** | -0.01 (0.02) | 0.45 | -0.01 (0.01) | 0.34 |
| Agitation x Time | -0.0005 (0.002) | 0.81 |  |  |
| Agitation x Time x Time | -0.00001 (0.00007) | 0.87 |  |  |
| **NPI affective** | -0.01 (0.02) | 0.59 | -0.007 (0.01) | 0.65 |
| Affective x Time | -0.005 (0.003) | 0.13 |  |  |
| Affective x Time x Time | 0.0001 (0.0001) | 0.17 |  |  |
| **NPI apathy** | -0.10 (0.04) | *0.02* | -0.06 (0.03) | *0.02* |
| Apathy x Time | -0.002 (0.006) | 0.71 |  |  |
| Apathy x Time x Time | 0.0001 (0.0002) | 0.60 |  |  |
| **SCUEQS** | 0.10 (0.03) | *0.005* | 0.05 (0.03) | 0.051 |
| SCUEQS x Time | -0.002 (0.004) | 0.68 |  |  |
| SCUEQS x Time x Time | 0.00003 (0.0001) | 0.76 |  |  |
| **Unit size** | -0.10 (0.04) | 0.01 | -0.05 (0.03) | 0.16 |
| Unit size x Time | 0.002 (0.004) | 0.61 |  |  |
| Unit size x Time x Time | 0.00002 (0.0001) | 0.90 |  |  |
| **Staff-to-resident ratio** | 2.80 (1.85) | 0.13 | 0.28 (1.35) | 0.84 |
| Staff-to-resident ratio x Time | -0.35 (0.20) | 0.08 |  |  |
| Staff-to-resident ratio x Time x Time | 0.01 (0.006) | 0.12 |  |  |
| SPPB, Short Physical Performance Battery.  ^a^ Based on the number of residents assessed with SPPB sum at the 7 time points. Cases with at least one missing value on covariates were excluded. (n_0_=482, n_6_=339, n_12_=278, n_18_=205, n_24_=159, n_30_=117, n_36_=91). ICC =26.4%.  SE, Standard Error; GMHR, General Medical Health Rating (dichotomized excellent/good versus fair/poor); CDR-sob, Clinical Dementia Rating sum of boxes; MOBID-2, Mobilization-Observation-Behaviour-Intensity-Dementia Pain Scale Part 1 and Part 2; NPI, Neuropsychiatric Inventory psychosis (delusions, hallucinations), agitation (agitation/aggression, disinhibition, irritability) and affective (depression, anxiety); SCUEQS, Special Care Unit Environmental Quality Scale. | | | | |

| **Supplementary Table S3** Nominal regression model for hierarchical data with group-belonging as outcome variable, Poor as reference, N=482^a^ | | | | |
| --- | --- | --- | --- | --- |
|  | | | | |
| **Characteristic** | **Bivariate model** | | **Multiple model** | |
|  | **OR (95% CI)** | **p-value** | **OR (95% CI)** | **p-value** |
| **Age** |  |  |  |  |
| Moderate | 1.00 (0.97; 1.03) | 0.90 | 1.00 (0.96; 1.03) | 0.75 |
| Good | 0.94 (0.91; 0.98) | *0.003* | 0.93 (0.89; 0.97) | *0.001* |
| **Sex** (male) |  |  |  |  |
| Moderate | 0.96 (0.64; 1.44) | 0.84 | 1.04 (0.65; 1.66) | 0.88 |
| Good | 0.68 (0.37; 1.72) | 0.22 | 0.62 (0.31; 1.25) | 0.18 |
| **GMHR** (good) |  |  |  |  |
| Moderate | 2.22 (1.48; 3.34) | *<0.001* | 1.69 (1.22; 3.16) | *0.006* |
| Good | 7.00 (3.63; 13.52) | *<0.001* | 5.44 (2.62; 11.31) | *<0.001* |
| **Medication** |  |  |  |  |
| Moderate | 1.01 (0.95; 1.08) | 0.68 | 1.04 (0.96; 1.12) | 0.32 |
| Good | 0.90 (0.82; 1.00) | 0.052 | 0.98 (0.87; 1.09) | 0.68 |
| **CDR-sob** |  |  |  |  |
| Moderate | 0.87 (0.82; 0.93) | *<0.001* | 0.91 (0.84; 0.97) | *0.007* |
| Good | 0.84 (0.77; 0.92) | *<0.001* | 0.83 (0.75; 0.92) | *<0.001* |
| **MOBID-2 Part 1** |  |  |  |  |
| Moderate | 0.92 (0.89; 0.95) | *<0.001* | 0.94 (0.90; 0.98) | *0.001* |
| Good | 0.84 (0.79; 0.90) | *<0.001* | 0.86 (0.79; 0.93) | *<0.001* |
| **MOBID-2 Part 2** |  |  |  |  |
| Moderate | 0.94 (0.90; 0.98) | *0.003* | 1.00 (0.95; 1.05) | 0.92 |
| Good | 0.92 (0.86; 0.98) | *0.02* | 1.03 (0.95; 1.12) | 0.47 |
| **NPI psychosis** |  |  |  |  |
| Moderate | 0.94 (0.89; 0.99) | *0.02* | 1.00 (0.93; 1.06) | 0.88 |
| Good | 1.00 (0.94; 1.07) | 0.98 | 1.08 (0.99; 1.17) | 0.08 |
| **NPI agitation** |  |  |  |  |
| Moderate | 0.94 (0.91; 0.97) | *<0.001* | 0.95 (0.91; 0.99) | *0.01* |
| Good | 0.97 (0.93; 1.01) | 0.14 | 0.96 (0.91; 1.01) | 0.09 |
| **NPI affective** |  |  |  |  |
| Moderate | 0.96 (0.93; 0.99) | *0.02* | 1.00 (0.96; 1.04) | 0.94 |
| Good | 1.00 (0.96; 1.05) | 0.89 | 1.04 (0.99; 1.10) | 0.14 |
| **NPI apathy** |  |  |  |  |
| Moderate | 0.88 (0.81; 0.95) | *0.001* | 0.93 (0.85; 1.01) | 0.08 |
| Good | 0.86 (0.76; 0.79) | *0.01* | 0.92 (0.81; 1.05) | 0.23 |
| **SCUEQS** |  |  |  |  |
| Moderate | 1.05 (1.00; 1.09) | *0.049* | 1.04 (0.99; 1.09) | 0.15 |
| Good | 1.08 (1.00; 1.15) | *0.046* | 1.07 (0.99; 1.15) | 0.09 |
| **Unit size** |  |  |  |  |
| Moderate | 0.96 (0.92; 1.00) | *0.048* | 0.95 (0.90; 1.00) | 0.054 |
| Good | 0.97 (0.91; 1.04) | 0.42 | 0.98 (0.92; 1.05) | 0.61 |
| **Staff-to-resident ratio** |  |  |  |  |
| Moderate | 1.62 (0.17; 15.78) | 0.68 | 1.7 (0.11; 29.65) | 0.70 |
| Good | 0.33 (0.01; 20.18) | 0.60 | 0.06 (0.00; 5.95) | 0.23 |
| ^a^ Cases with at least one missing value on covariates were excluded (Poor n=176, Moderate n=230, Good n=76).  GMHR, General Medical Health Rating (dichotomized excellent/good versus fair/poor); CDR-sob, Clinical Dementia Rating sum of boxes; MOBID-2, Mobilization-Observation-Behaviour-Intensity-Dementia Pain Scale Part 1 and Part 2; NPI, Neuropsychiatric Inventory psychosis (delusions, hallucinations), agitation (agitation/aggression, disinhibition, irritability) and affective (depression, anxiety); SCUEQS, Special Care Unit Environmental Quality Scale. | | | | |

| **Supplementary Table S4** Individual characteristics at follow-up | | | | | | |
| --- | --- | --- | --- | --- | --- | --- |
| **Characteristic** | **T_6_ (n=437)** | **T_12_ (n=374)** | **T_18_ (n=307)** | **T_24_ (n=260)** | **T_30_ (n=209)** | **T_36_ (n=170)** |
| **SPPB**, n | 327 | 305 | 238 | 194 | 161 | 133 |
| mean (SD) | 4.0 (3.6) | 3.6 (3.5) | 3.2 (3.5) | 2.6 (3.3) | 2.5 (3.4) | 2.0 (2.9) |
| **GMHR Good,** n | 245 | 362 | 292 | 245 | 186 | 147 |
| n (%) | 207(48.7) | 145(40.1) | 100 (34.2) | 79 (32.2) | 50 (26.9) | 40 (27.2) |
| **Medication**, n | 437 | 374 | 307 | 260 | 209 | 170 |
| mean (SD) | 6.2 (3.0) | 6.0 (3.0) | 5.9 (3.3) | 6.2 (3.3) | 6.3 (3.3) | 6.2 (3.7) |
| **CDR-sob**, n | 431 | 369 | 304 | 256 | 199 | 165 |
| mean (SD) | 11.8 (3.8) | 12.4 (3.7) | 13.1 (3.7) | 13.7 (3.7) | 14.3 (3.4) | 14.4 (3.5) |
| **MOBID-2 Part 1**, n | 424 | 355 | 293 | 235 | 186 | 142 |
| mean (SD) | 4.8 (6.5) | 5.8 (7.4) | 5.6 (6.6) | 6.3 (8.5) | 6.6 (7.7) | 7.4 (8.9) |
| **MOBID-2 Part 2**, n | 422 | 353 | 292 | 236 | 186 | 142 |
| mean (SD) | 3.4 (4.9) | 3.5 (4.8) | 3.7 (5.2) | 3.4 (4.5) | 3.1 (4.2) | 3.9 (4.8) |
| **NPI psychosis**, n | 430 | 369 | 306 | 255 | 201 | 163 |
| mean (SD) | 2.4 (4.5) | 2.2 (3.9) | 2.7 (4.6) | 2.8 (4.7) | 2.5 (4.0) | 2.7 (4.4) |
| **NPI agitation**, n | 430 | 370 | 305 | 254 | 202 | 164 |
| mean (SD) | 5.0 (7.7) | 5.7 (8.3) | 6.0 (8.0) | 7.8 (9.5) | 7.1 (9.1) | 7.4 (9.0) |
| **NPI affective**, n | 433 | 369 | 305 | 254 | 203 | 165 |
| mean (SD) | 3.8 (5.8) | 3.7 (5.4) | 4.3 (5.8) | 4.0 (5.4) | 3.8 (5.4) | 3.5 (5.3) |
| **NPI apathy**, n | 432 | 369 | 306 | 255 | 203 | 163 |
| mean (SD) | 1.1 (2.6) | 1.2 (2.5) | 1.5 (2.8) | 1.8 (3.2) | 1.9 (3.4) | 1.8 (3.1) |
| SPPB, The Short Physical Performance Battery (0-12); SD, Standard Deviation; GMHR, General Medical Health Rating (dichotomized excellent/good versus fair/poor); CDR-sob, Clinical Dementia Rating sum of boxes (0-18); MOBID-2, Mobilization-Observation-Behaviour-Intensity-Dementia Pain Scale Part 1 (0-50) and Part 2 (0-50); NPI, Neuropsychiatric Inventory psychosis (delusions, hallucinations, 0-24), agitation (agitation/aggression, disinhibition, irritability, 0-36), affective (depression, anxiety, 0-24) and apathy (apathy, 0-12). | | | | | | |
